# UDEC-MO: an uncertainty-guided deep embedded clustering framework for bulk and single-cell multi-omics data

**DOI:** 10.1093/bib/bbag435

**Published:** 2026-08-10

**Authors:** Jiawei Li, Taoyuan Ye, Yilang Xiao, Mengyuan Zhao, Limin Jiang, Shizhan Chen, Fei Guo, Jijun Tang

**Affiliations:** School of Computer Science and Technology, College of Intelligence and Computing, Tianjin University, No. 135 Yaguan Road, Jinnan District, Tianjin 300350, China; Faculty of Computer Science and Artificial Intelligence, Shenzhen University of Advanced Technology, No. 1 Gongchang Road, Guangming District, Shenzhen, Guangdong 518107, China; Faculty of Computer Science and Artificial Intelligence, Shenzhen University of Advanced Technology, No. 1 Gongchang Road, Guangming District, Shenzhen, Guangdong 518107, China; Faculty of Computer Science and Artificial Intelligence, Shenzhen University of Advanced Technology, No. 1 Gongchang Road, Guangming District, Shenzhen, Guangdong 518107, China; Shenzhen Institute of Advanced Technology, Chinese Academy of Sciences, No. 1068 Xueyuan Avenue, Nanshan District, Shenzhen, Guangdong 518055, China; Department of Public Health Sciences, University of Miami, 1120 NW 14th Street, CRB 1051, Miami, FL 33136, United States; School of Computer Science and Technology, College of Intelligence and Computing, Tianjin University, No. 135 Yaguan Road, Jinnan District, Tianjin 300350, China; School of Computer Science and Engineering, Central South University, Computer Building, No. 932 South Lushan Road, Yuelu District, Changsha, Hunan 410083, China; Faculty of Computer Science and Artificial Intelligence, Shenzhen University of Advanced Technology, No. 1 Gongchang Road, Guangming District, Shenzhen, Guangdong 518107, China; Shenzhen Institute of Advanced Technology, Chinese Academy of Sciences, No. 1068 Xueyuan Avenue, Nanshan District, Shenzhen, Guangdong 518055, China

**Keywords:** multi-omics clustering, multi-level dynamic uncertainty, uncertainty-aware reconstruction, deep embedded clustering

## Abstract

Bulk and single-cell multi-omics technologies provide complementary molecular views for characterizing disease heterogeneity and cellular diversity. However, robust multi-omics clustering remains challenging due to high dimensionality, pervasive noise, modality heterogeneity, and substantial reliability variation across features, modalities, and samples or cells. Existing clustering methods often insufficiently account for such multilevel data quality variation. Here, we present **UDEC-MO**, an **U**ncertainty-guided **D**eep **E**mbedded **C**lustering framework for robust **M**ulti-**O**mics clustering. UDEC-MO first estimates feature-wise heteroscedastic uncertainty through uncertainty-aware reconstruction and summarizes it into modality-level and instance-level uncertainty scores. The instance-level uncertainty is further transformed into reliability weights to modulate the Kullback–Leibler-divergence loss in deep embedded clustering, allowing reliable samples or cells to guide cluster refinement while reducing the influence of highly uncertain instances. We evaluated UDEC-MO on both bulk cancer multi-omics datasets and single-cell multi-omics datasets generated by different sequencing technologies. The results demonstrate that UDEC-MO achieves competitive or superior clustering performance across multiple metrics and provides uncertainty-derived reliability indicators that may offer auxiliary information for characterizing potentially unreliable features, less reliable modalities, and ambiguous instances.

## Introduction

The rapid advancement of high-throughput sequencing technologies has enabled the simultaneous profiling of diverse molecular modalities at both bulk and single-cell levels, including transcriptomics, epigenomics, proteomics, and so on [1–5]. This has led to the accumulation of large-scale multi-omics datasets, offering unprecedented opportunities to dissect complex biological systems, identify disease subtypes, and characterize potential cell types [6–9]. However, multi-omics clustering remains challenging because omics profiles are often high-dimensional, sparse, heterogeneous, and noisy, with substantial variation in data quality across modalities and samples [10–13]. Bulk multi-omics data are often affected by batch effects, platform-specific biases, and cohort-level heterogeneity, whereas single-cell multi-omics data are further challenged by dropout events, low molecular capture efficiency, and uneven coverage across modalities. These factors can induce substantial reliability variation across features, modalities, and samples or cells, thereby compromising robust multi-omics clustering.

Effective multi-omics clustering first requires a discriminative and robust embedding space that can preserve biologically meaningful structures while reducing the influence of technical noise and modality heterogeneity. To this end, many existing multi-omics embedding learning methods rely on reconstruction [14–16], where neural networks are trained to recover the original multi-omics profiles from low-dimensional latent representations. Representative approaches include Autoencoders (AEs) [17, 18], Denoising Autoencoders (DAEs) [19, 20], and Variational Autoencoders (VAEs) [21, 22]. For single-cell data, reconstruction objectives based on the Zero-Inflated Negative Binomial (ZINB) distribution have further been introduced to account for dropout events and over-dispersion in single-cell RNA sequencing (scRNA-seq) count data [23–25]. Related studies have also demonstrated the value of deep learning, multi-omics integration, representation learning, and heterogeneity quantification in disease subtyping and cellular state analysis [26–29].

Recent advanced methods have further promoted multi-omics integration and clustering from different perspectives. MOFA and MOFA+ [30, 31] formulate multi-omics integration as a probabilistic factor analysis problem, learning interpretable low-dimensional factors that capture shared and modality-specific sources of variation across omics layers. MOSA [32] adopts a deep generative strategy to synthesize missing molecular profiles and improve the completeness of multi-omics representations. For single-cell multi-omics data, scMDC [33] combines ZINB-based deep representation learning with clustering optimization to jointly learn latent embeddings and cell clusters, while Matilda [34] provides a neural multitask framework for multimodal single-cell omics analysis. More recently, scMHNN [35] employs hypergraph neural networks and contrastive learning to model high-order relationships across transcriptomic, epigenomic, and proteomic modalities, and scMNMF [36] introduces a joint nonnegative matrix factorization framework to couple cell clustering with feature selection.

**Figure 1:**
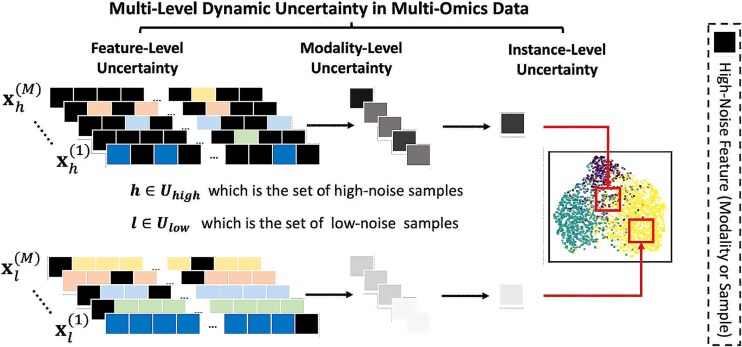
Multi-level dynamic data uncertainty in multi-omics data, showing heterogeneous noise and reliability variation across feature, modality, and instance levels, where black squares denote noisy features, modalities, or instances corresponding to high data uncertainty.

However, these methods often neglect the dynamic uncertainty across features, modalities, and samples or cells, which limits their robustness when handling heterogeneous and noisy multi-omics data. As illustrated in Fig. 1, bulk and single-cell multi-omics datasets commonly exhibit multi-level dynamic data uncertainty caused by sequencing imperfections, technical biases, biological stochasticity, and ambiguous biological states. This uncertainty manifests hierarchically, progressing from individual features to modality-level reliability and further to instance-level reliability, ultimately affecting the quality of learned representations. Currently, uncertainty estimation has been widely applied in various fields, allowing for the quantification of data uncertainty and facilitating more robust decision-making and model interpretation [37–39]. Nevertheless, how to explicitly model multi-level data uncertainty and further utilize the estimated reliability to guide multi-omics clustering remains insufficiently explored.

To address these challenges, we propose **UDEC-MO**, an **U**ncertainty-guided **D**eep **E**mbedded **C**lustering framework for robust **M**ulti-**O**mics clustering across both bulk and single-cell settings. UDEC-MO follows a two-stage optimization strategy that integrates uncertainty-aware reconstruction (UAR) with reliability-weighted cluster refinement. In the first stage, UDEC-MO estimates feature-wise heteroscedastic uncertainty during multi-omics representation learning and summarizes it into modality-level and instance-level uncertainty scores, enabling multi-level characterization of data quality variation across features, modalities, and samples or cells. In the second stage, the instance-level uncertainty is transformed into reliability weights to modulate the Kullback–Leibler (KL)-divergence loss between the auxiliary target distribution and soft cluster assignments, allowing reliable samples or cells to guide cluster refinement while reducing the influence of highly uncertain instances. Unlike existing methods, which mainly focus on cross-modal fusion, latent representation learning, or graph-based association modeling, UDEC-MO explicitly characterizes reliability variations across features, modalities, and instances, and further uses instance-level reliability to guide clustering optimization. We evaluate UDEC-MO on both bulk cancer multi-omics datasets from TCGA [2] and single-cell multi-omics datasets generated by different sequencing technologies [3–5]. Experimental results demonstrate that UDEC-MO achieves competitive or superior clustering performance across multiple metrics and provides meaningful uncertainty estimates for identifying unreliable features, less reliable modalities, and ambiguous samples or cells.

## Materials and methods

### Bulk and single-cell multi-omics datasets

In this study, we evaluate UDEC-MO on publicly available bulk and single-cell multi-omics datasets obtained from previous studies [35, 40], covering diverse biological contexts and sequencing technologies. For bulk cancer multi-omics analysis, we use three patient-level TCGA datasets curated by Wang *et al*. [40], including BRCA (breast cancer), KIPAN (pan-kidney cancer), and LGG (low-grade glioma). Each dataset contains matched messenger RNA (mRNA) expression, DNA methylation, and miRNA expression profiles. For single-cell multi-omics analysis, we use three publicly available tri-modal datasets collected and processed by Li *et al*. [35], including DOGMA-seq, TEA-seq, and NEAT-seq. These datasets provide paired chromatin accessibility, gene expression, and protein abundance measurements from the same cells. Specifically, DOGMA-seq [41] was profiled from human PBMCs and includes 13 763 cells across 25 cell types. TEA-seq [42] was also profiled from human PBMCs and consists of 25 517 cells across 12 immune-related cell types. NEAT-seq [43] profiles human CD4 memory T cells and covers seven distinct T cell subtypes. All data processing steps followed previous studies [35, 40]. Detailed information on the bulk and single-cell multi-omics datasets is provided in Supplementary Tables S1 and S2, respectively.

### UDEC-MO framework

**Figure 2:**
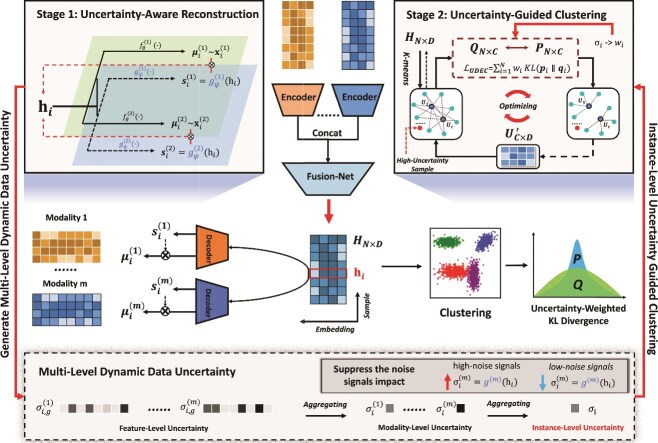
Framework of **UDEC-MO**. Modality-specific encoders first process multi-omics data to obtain embeddings for each omics type, which are then integrated into a shared representation via an attention-based fusion network. UDEC-MO follows a two-stage optimization strategy. **Stage 1: Uncertainty-Aware Reconstruction**, where the shared embedding is decoded by modality-specific reconstruction networks to produce reconstructed values and feature-wise heteroscedastic uncertainty estimates. These uncertainty estimates are further aggregated into modality-level and instance-level uncertainty scores, enabling multi-level characterization of data reliability. **Stage 2: Uncertainty-Guided Deep Embedded Clustering**, where instance-level uncertainty is transformed into reliability weights to modulate the KL-divergence loss between the auxiliary target distribution and soft cluster assignments, allowing reliable samples or cells to contribute more to cluster refinement while reducing the influence of uncertain instances.

As shown in Fig. 2, UDEC-MO consists of a multi-omics fusion module and two sequential optimization stages. The multi-omics fusion module learns a shared representation from modality-specific embeddings, providing a unified latent space for subsequent optimization. Based on this representation, the first stage estimates feature-wise heteroscedastic uncertainty and aggregates it into modality-level and instance-level reliability information. The second stage further uses instance-level reliability to weight the deep embedded clustering (DEC) objective, thereby reducing the influence of uncertain samples or cells during cluster refinement. This design allows UDEC-MO to jointly improve representation robustness and clustering reliability. Below, we describe each component in detail.

#### Multi-omics fusion

Given a multi-omics dataset with $N$ instances and $M$ modalities, the input of the $i$th instance is denoted as $\{\mathbf{x}_{i}^{(m)}\}_{m=1}^{M}$, where $\mathbf{x}_{i}^{(m)} \in \mathbb{R}^{G_{m}}$ represents the feature vector of the $m$th modality. The goal of the fusion module is to learn a unified embedding $\mathbf{h}_{i} \in \mathbb{R}^{D}$ that integrates complementary information across modalities.

For each modality, we first employ a modality-specific encoder to project the input into a latent space:


(1) \begin{eqnarray*}& \mathbf{z}_{i}^{(m)} = \phi_{\mathrm{enc}}^{(m)}(\mathbf{x}_{i}^{(m)}),\end{eqnarray*}

where $\phi _{\mathrm{enc}}^{(m)}(\cdot )$ denotes a two-layer multilayer perceptron (MLP) encoder. The modality-specific embeddings are then stacked as $\mathbf{Z}_{i} = [\mathbf{z}_{i}^{(1)}, \dots , \mathbf{z}_{i}^{(M)}] \in \mathbb{R}^{M \times D_{z}}$.

To capture inter-modality dependencies, we apply an attention-based fusion mechanism [44]:


(2) \begin{eqnarray*}& \widetilde{\mathbf{Z}}_{i} = \mathrm{softmax}\!\left(\frac{\mathbf{Z}_{i}\mathbf{W}^{Q} (\mathbf{Z}_{i}\mathbf{W}^{K})^{\top}}{\sqrt{d_{k}}}\right)\mathbf{Z}_{i}\mathbf{W}^{V},\end{eqnarray*}

where $\mathbf{W}^{Q}$, $\mathbf{W}^{K}$, and $\mathbf{W}^{V}$ are learnable projection matrices, and $\widetilde{\mathbf{z}}_{i}^{(m)}$ denotes the attended representation of the $m$th modality.

Finally, the attended modality representations are concatenated and passed through a fusion projection network to obtain the unified embedding:


(3) \begin{eqnarray*}& \mathbf{h}_{i} = \phi_{\mathrm{fus}}\!\left(\mathrm{Concat}(\widetilde{\mathbf{z}}_{i}^{(1)}, \dots, \widetilde{\mathbf{z}}_{i}^{(M)})\right),\end{eqnarray*}

where $\phi _{\mathrm{fus}}(\cdot )$ is implemented as a two-layer MLP. The overall fused embedding matrix is denoted as $\mathbf{H} = [\mathbf{h}_{1}, \dots , \mathbf{h}_{N}]^{\top } \in \mathbb{R}^{N \times D}$, which is then used for subsequent UAR and uncertainty-guided DEC.

#### Uncertainty-aware reconstruction

Multi-omics data are often affected by heterogeneous noise sources, such as sequencing noise, dropout events, platform-specific biases, and instance-specific quality variation. These factors can induce substantial reliability variation across features, modalities, and samples or cells, posing challenges for robust representation learning and clustering. Inspired by modality-uncertainty modeling [39], UDEC-MO adopts a UAR objective to estimate feature-wise heteroscedastic uncertainty from the fused representation, thereby reducing the influence of unreliable signals on the learned embedding.

Given the fused embedding $\mathbf{h}_{i}$ of the $i$th instance, UDEC-MO reconstructs each modality through modality-specific reconstruction and uncertainty prediction networks. For the $m$th modality, the reconstructed mean vector and feature-wise log-variance are predicted as


(4) \begin{eqnarray*}& \boldsymbol{\mu}_{i}^{(m)} = f_{\theta}^{(m)}(\mathbf{h}_{i}), \quad \mathbf{s}_{i}^{(m)} = g_{\psi}^{(m)}(\mathbf{h}_{i}),\end{eqnarray*}

where $\boldsymbol{\mu }_{i}^{(m)} \in \mathbb{R}^{G_{m}}$ denotes the reconstructed mean vector, and $\mathbf{s}_{i}^{(m)} \in \mathbb{R}^{G_{m}}$ denotes the predicted log-variance. Both $f_{\theta }^{(m)}(\cdot )$ and $g_{\psi }^{(m)}(\cdot )$ are implemented as modality-specific two-layer MLPs that map latent embeddings back to the input feature space.

Under a heteroscedastic Gaussian assumption, each observed feature is modeled as


(5) \begin{eqnarray*}& x_{ig}^{(m)} \mid \mathbf{h}_{i} \sim \mathcal{N}\left(\mu_{ig}^{(m)},(\sigma_{ig}^{(m)})^{2}\right).\end{eqnarray*}

We adopt the heteroscedastic Gaussian formulation as a unified engineering choice for both bulk and single-cell multi-omics inputs after preprocessing and normalization. Although ZINB is well suited for raw scRNA-seq count modeling, the Gaussian likelihood provides a modality-agnostic objective that keeps the uncertainty term comparable across omics types. This choice does not imply that raw single-cell counts are intrinsically Gaussian; rather, it is used to estimate relative reconstruction uncertainty in the preprocessed multi-omics setting. Thus, the conditional likelihood of each observation can be written as


(6) \begin{eqnarray*}& \begin{aligned} p(x_{ig}^{(m)}|\mathbf{h}_{i}) &= \frac{1}{\sqrt{2\pi(\sigma_{ig}^{(m)})^{2}}} \exp\left(-\frac{(x_{ig}^{(m)}-\mu_{ig}^{(m)})^{2}}{2(\sigma_{ig}^{(m)})^{2}}\right) \\ &= \frac{1}{\sqrt{2\pi\exp(s_{ig}^{(m)})}} \exp\left(-\frac{(x_{ig}^{(m)}-\mu_{ig}^{(m)})^{2}}{2\exp(s_{ig}^{(m)})}\right), \end{aligned}\end{eqnarray*}

where the second equality follows from parameterizing the variance with the predicted log-variance $s_{ig}^{(m)}$. By taking the negative log-likelihood and omitting constant terms, we obtain


(7) \begin{eqnarray*}& \begin{gathered} -\log p(x_{ig}^{(m)}|\mathbf{h}_{i}) = \frac{1}{2}\left[ \frac{\left(x_{ig}^{(m)}-\mu_{ig}^{(m)}\right)^{2}}{\exp\left(s_{ig}^{(m)}\right)} +s_{ig}^{(m)} \right]. \end{gathered}\end{eqnarray*}

Assuming conditional independence across features, modalities, and instances given $\mathbf{h}_{i}$, the UAR loss is formulated as


(8) \begin{eqnarray*}& \begin{gathered} \mathcal{L}_{\mathrm{UAR}} = \sum_{i=1}^{N}\sum_{m=1}^{M}\frac{1}{G_{m}}\sum_{g=1}^{G_{m}}\frac{1}{2} \left[ \frac{\left(x_{ig}^{(m)}-\mu_{ig}^{(m)}\right)^{2}}{\exp\left(s_{ig}^{(m)}\right)} + s_{ig}^{(m)} \right]. \end{gathered}\end{eqnarray*}

Here, the first term is an uncertainty-weighted reconstruction error, where features with larger predicted uncertainty contribute less to the reconstruction penalty. The second term regularizes the predicted uncertainty and prevents the model from trivially assigning excessively large variance to all observations. Intuitively, when an observation is difficult to reconstruct because of noise or missing signal, the model can increase its predicted variance to reduce over-penalization; however, the log-variance penalty discourages indiscriminately inflating uncertainty. The balance between these two terms encourages high uncertainty only for observations whose reconstruction residuals remain large, allowing the learned variance to approximate heteroscedastic data reliability. For numerical stability, $s_{ig}^{(m)}$ is clipped into $[-10,10]$ before computing $\exp (s_{ig}^{(m)})$.

After optimization, the multi-level dynamics uncertainty is derived from the predicted log-variance. Specifically, the feature-, modality-, and instance-level uncertainties are defined as


(9) \begin{eqnarray*}& \begin{aligned} \sigma_{ig}^{(m)} &= \exp\left(\frac{1}{2}s_{ig}^{(m)}\right), \\ \sigma_{i}^{(m)} &= \frac{1}{G_{m}}\sum_{g=1}^{G_{m}}\sigma_{ig}^{(m)}, \\ \sigma_{i} &= \sum_{m=1}^{M}\sigma_{i}^{(m)}. \end{aligned}\end{eqnarray*}

Here, $\sigma _{ig}^{(m)}$ corresponds to the predicted standard deviation of the $g$th feature in the $m$th modality for the $i$th instance, capturing feature-level uncertainty. The modality-level uncertainty $\sigma _{i}^{(m)}$ is obtained by averaging feature-level uncertainties within each modality, reflecting the overall reliability of each omics view. The instance-level uncertainty $\sigma _{i}$ is further obtained by aggregating modality-level uncertainties across all modalities, representing the overall data quality of each sample or cell. A larger $\sigma _{i}$ indicates higher accumulated uncertainty and lower reliability for clustering refinement.

#### Uncertainty-guided deep embedded clustering

After UAR, UDEC-MO obtains the fused embeddings $\mathbf{H}=\{\mathbf{h}_{i}\}_{i=1}^{N}$ and the instance-level uncertainties $\boldsymbol{\sigma }=\{\sigma _{i}\}_{i=1}^{N}$ for samples or cells. Instead of using uncertainty only for post hoc interpretation, we incorporate it into the DEC [45] objective to guide cluster refinement, resulting in an uncertainty-guided deep embedded clustering module (UDEC).

Following the DEC framework, we first initialize $C$ cluster centroids $\mathbf{U}=\{\mathbf{u}_{j}\}_{j=1}^{C}$ by applying $k$-means to the learned embeddings $\mathbf{H}$. Given an embedding $\mathbf{h}_{i}$ and a cluster centroid $\mathbf{u}_{j}$, the soft assignment of instance $i$ to cluster $j$ is computed using Student’s $t$-distribution:


(10) \begin{eqnarray*}& q_{ij} = \frac{\left(1+\|\mathbf{h}_{i}-\mathbf{u}_{j}\|^{2}/\alpha\right)^{-\frac{\alpha+1}{2}}} {\sum_{j^{\prime}=1}^{C}\left(1+\|\mathbf{h}_{i}-\mathbf{u}_{j^{\prime}}\|^{2}/\alpha\right)^{-\frac{\alpha+1}{2}}},\end{eqnarray*}

where $\alpha$ is the degrees-of-freedom parameter, which is set to $1$ by default. The soft assignment matrix is denoted as $\mathbf{Q}=[q_{ij}]\in \mathbb{R}^{N\times C}$.

To sharpen high-confidence assignments and reduce the influence of large clusters, an auxiliary target distribution is constructed as


(11) \begin{eqnarray*}& p_{ij} = \frac{q_{ij}^{2}/f_{j}}{\sum_{j^{\prime}=1}^{C}q_{ij^{\prime}}^{2}/f_{j^{\prime}}}, \quad f_{j}=\sum_{i=1}^{N}q_{ij},\end{eqnarray*}

where $f_{j}$ denotes the soft cluster frequency. The target distribution matrix is denoted as $\mathbf{P}=[p_{ij}]\in \mathbb{R}^{N\times C}$.

In standard DEC, all instances contribute equally to the KL-divergence loss between $\mathbf{P}$ and $\mathbf{Q}$. However, in noisy multi-omics data, highly uncertain samples or cells may produce unstable assignments and negatively affect cluster refinement. To address this issue, UDEC-MO converts the instance-level uncertainty $\sigma _{i}$ into a reliability weight:


(12) \begin{eqnarray*}& w_{i} = \frac{\exp(-\sigma_{i}/\tau)} {\sum_{i^{\prime}=1}^{N}\exp(-\sigma_{i^{\prime}}/\tau)},\end{eqnarray*}

where $\tau$ is the temperature parameter controlling the sharpness of uncertainty-based weighting. A smaller $\tau$ assigns much lower weights to uncertain instances, whereas a larger $\tau$ makes the weighting scheme closer to unweighted. In our implementation, we set $\tau =1$.

The final uncertainty-guided clustering loss is defined as


(13) \begin{eqnarray*} \mathcal{L}_{\mathrm{UDEC}} &= \sum_{i=1}^{N} w_{i} \, \mathrm{KL}(\mathbf{p}_{i} \| \mathbf{q}_{i}) \nonumber \\ &= \sum_{i=1}^{N} w_{i} \sum_{j=1}^{C} p_{ij}\log\frac{p_{ij}}{q_{ij}}. \end{eqnarray*}

Compared with the original DEC objective, $\mathcal{L}_{\mathrm{UDEC}}$ explicitly incorporates instance-level reliability into clustering optimization. Instances with lower uncertainty receive larger weights and provide stronger guidance for refining cluster structures, whereas highly uncertain instances are down-weighted to reduce their negative impact on clustering.

#### Model optimization and implementation details

UDEC-MO is implemented in PyTorch and trained with the Adam optimizer on a workstation equipped with an NVIDIA RTX A6000 GPU. The model is optimized through two consecutive stages. The overall training procedure is summarized in Supplementary Algorithm S1, and the implementation is available at https://github.com/ljw-struggle/UDEC-MO.

*Stage 1: Uncertainty-aware reconstruction.* We first train the multi-omics fusion module and reconstruction networks using a standard mean squared reconstruction objective for 200 epochs with a learning rate of $5 \times 10^{-3}$ to obtain a stable embedding space. Starting from this pretrained model, we further optimize the fusion module, reconstruction networks, and uncertainty prediction networks by minimizing $\mathcal{L}_{\mathrm{UAR}}$ for 100 epochs with a learning rate of $1 \times 10^{-3}$. After this stage, feature-level uncertainty $\sigma _{ig}^{(m)}$, modality-level uncertainty $\sigma _{i}^{(m)}$, and instance-level uncertainty $\sigma _{i}$ are derived from the uncertainty estimation scheme described above.

*Stage 2: Uncertainty-guided clustering optimization.* The cluster centroids $\mathbf{U}$ are initialized by applying $k$-means to the learned embeddings $\mathbf{H}$. During clustering optimization, $\mathbf{Q}$ and $\mathbf{P}$ are updated at each epoch, corresponding to an update period of 1. The embedding network and cluster centroids are jointly optimized by minimizing $\mathcal{L}_{\mathrm{UDEC}}$, where the instance-level uncertainties obtained from Stage 1 are fixed and used to construct reliability weights. In this stage, the initial learning rate is set to $1 \times 10^{-3}$, and a step decay scheduler reduces the learning rate by a factor of 0.9 every 20 epochs. The maximum number of clustering epochs is set to 100, and the optimization stops early when the cluster assignment change ratio is smaller than $0.1\%$.

*Computational cost and scalability.* The computational cost of UDEC-MO is mainly determined by the modality-specific encoders/decoders and the DEC-style clustering refinement. For a dataset with $N$ instances, $M$ modalities, and processed feature dimensions $\{G_{m}\}_{m=1}^{M}$, the reconstruction stage scales approximately linearly with $N$ and the total processed feature dimension $\sum _{m} G_{m}$, while the clustering stage scales with $N C D$ for $C$ clusters and embedding dimension $D$. In this study, the largest evaluated dataset is TEA-seq with 25 517 cells. For substantially larger single-cell atlases, mini-batch training, periodic centroid updates, and feature preselection can be used to reduce memory and runtime costs without changing the core objective.

## Results

### Baseline methods

To comprehensively evaluate the effectiveness of UDEC-MO on clustering, we compare it with representative baseline methods covering different multi-omics integration and clustering paradigms. These baselines include reconstruction-based embedding methods, probabilistic factor analysis methods, deep generative integration methods, matrix factorization-based clustering methods, and recent single-cell multi-omics clustering frameworks. For reconstruction-based baselines, AE, DAE, and VAE adopt encoder–decoder architectures similar to the reconstruction module of UDEC-MO, where different omics modalities are encoded into a shared latent representation and then reconstructed by modality-specific decoders. For representative multi-omics integration methods, MOFA [30, 31], MOSA [32], scMDC [33], Matilda [34], scMHNN [35], and scMNMF [36] are included following their original designs. These methods cover both bulk and single-cell multi-omics settings and provide diverse comparisons for assessing the clustering performance of UDEC-MO. To further broaden the benchmark coverage, we additionally evaluated Cobolt [46], scGLUE [47], Mowgli [48], and MultiMAP [49]. Detailed descriptions of the baseline methods are provided in Supplementary Table S3.

### Evaluation metrics

We evaluate the clustering performance of UDEC-MO using four widely adopted metrics, including Clustering Accuracy (**ACC**), Normalized Mutual Information (**NMI**), Adjusted Rand Index (**ARI**), and Average Silhouette Width (**ASW**). Among them, ACC, NMI, and ARI are external metrics that assess the agreement between predicted cluster assignments and ground-truth labels, while ASW is an internal metric that evaluates the geometric quality of the learned embedding space.

Let $\mathbf{y}=\{y_{i}\}_{i=1}^{N}$ denote the ground-truth labels and $\hat{\mathbf{y}}=\{\hat{y}_{i}\}_{i=1}^{N}$ denote the predicted cluster assignments. ACC is computed as


(14) \begin{eqnarray*}& \mathrm{ACC}=\frac{1}{N}\sum_{i=1}^{N}\mathbb{I}\left(y_{i}=\pi(\hat{y}_{i})\right),\end{eqnarray*}

where $\pi (\cdot )$ denotes the optimal one-to-one mapping obtained by the Hungarian algorithm, and $\mathbb{I}(\cdot )$ is the indicator function.

NMI measures the shared information between clustering assignments and ground-truth labels:


(15) \begin{eqnarray*}& \mathrm{NMI}(\hat{\mathbf{y}},\mathbf{y})=\frac{2I(\hat{\mathbf{y}};\mathbf{y})}{H(\hat{\mathbf{y}})+H(\mathbf{y})},\end{eqnarray*}

where $I(\cdot ;\cdot )$ denotes mutual information and $H(\cdot )$ denotes entropy.

ARI evaluates clustering similarity while correcting for chance:


(16) \begin{eqnarray*}& \mathrm{ARI}=\frac{\mathrm{RI}-\mathbb{E}[\mathrm{RI}]}{\max(\mathrm{RI})-\mathbb{E}[\mathrm{RI}]},\end{eqnarray*}

where $\mathrm{RI}$ denotes the Rand Index and $\mathbb{E}[\mathrm{RI}]$ is its expected value under random assignments.

ASW is an internal metric computed based on the predicted cluster assignments $\hat{\mathbf{y}}$ and evaluates the geometric structure of the learned embedding space, including intra-cluster cohesion and inter-cluster separation. For each instance $i$, the silhouette coefficient is defined as


(17) \begin{eqnarray*}& \rho_{i}=\frac{b_{i}-a_{i}}{\max(a_{i},b_{i})},\end{eqnarray*}

where $a_{i}$ is the average distance between instance $i$ and other instances in the same predicted cluster, and $b_{i}$ is the minimum average distance between instance $i$ and instances in other predicted clusters. The ASW score is computed as


(18) \begin{eqnarray*}& \mathrm{ASW}=\frac{1}{N}\sum_{i=1}^{N}\rho_{i}.\end{eqnarray*}

ACC, NMI, and ARI are bounded within the interval $[0,1]$, where higher values indicate better agreement with ground-truth labels. ASW is theoretically bounded within $[-1,1]$; however, in our experiments all ASW values are nonnegative. For ease of comparison, all metrics are reported as percentages (0%–100%). All metric implementations are based on the scikit-learn library [50].

### Clustering performance comparison

We evaluate the clustering performance of UDEC-MO on both bulk and single-cell multi-omics datasets. The results are summarized in Tables 1 and 2, with additional benchmark results reported in Supplementary Table S4. Overall, UDEC-MO demonstrates strong and stable performance across different datasets, particularly on external clustering metrics, including ACC, NMI, and ARI.

**Table 1:** Clustering performance comparison of different methods across different bulk multi-omics datasets. All results are reported as mean $\pm$ standard deviation over 10 independent runs, with values rounded to one decimal place. The best results are highlighted in bold.

		Method									
Dataset	Metric	AE	DAE	VAE	MOFA	MOSA	scMDC	Matilda	scMHNN	scMNMF	UDECMO
KIPAN	ACC	65.3$\pm$3.9	69.9$\pm$5.5	71.8$\pm$5.6	72.1$\pm$0.3	77.8$\pm$1.0	80.9$\pm$2.5	73.5$\pm$0.9	79.3$\pm$0.3	72.3$\pm$1.2	**82.0$\pm$1.9**
	NMI	38.3$\pm$2.8	41.6$\pm$2.9	39.4$\pm$3.5	30.4$\pm$0.6	34.8$\pm$1.0	48.0$\pm$4.0	44.4$\pm$2.0	45.8$\pm$0.4	40.9$\pm$1.7	**51.9$\pm$2.0**
	ARI	30.1$\pm$3.0	34.6$\pm$3.6	38.9$\pm$4.4	25.7$\pm$0.6	30.8$\pm$0.7	50.1$\pm$3.0	45.1$\pm$3.0	44.3$\pm$1.1	38.5$\pm$2.4	**55.6$\pm$2.4**
	ASW	51.9$\pm$2.2	50.3$\pm$3.8	48.5$\pm$4.4	12.7$\pm$1.2	14.8$\pm$0.1	52.4$\pm$3.5	49.4$\pm$4.3	49.9$\pm$1.0	24.6$\pm$1.6	**56.6$\pm$2.6**
BRCA	ACC	60.2$\pm$1.0	61.1$\pm$1.6	51.6$\pm$5.3	61.3$\pm$1.5	61.5$\pm$0.5	61.8$\pm$1.4	56.4$\pm$4.8	58.2$\pm$0.5	52.4$\pm$1.2	**64.0$\pm$1.8**
	NMI	35.5$\pm$1.4	36.9$\pm$1.1	30.1$\pm$4.3	38.3$\pm$2.6	35.4$\pm$0.6	40.6$\pm$1.9	36.6$\pm$3.4	34.0$\pm$0.7	31.9$\pm$1.2	**41.8$\pm$2.8**
	ARI	28.7$\pm$2.2	28.3$\pm$2.7	20.2$\pm$4.8	29.7$\pm$5.4	24.5$\pm$0.5	28.8$\pm$1.4	25.2$\pm$4.7	24.8$\pm$0.4	21.3$\pm$1.2	**30.4$\pm$2.0**
	ASW	38.2$\pm$2.3	35.3$\pm$3.1	30.6$\pm$3.4	10.5$\pm$0.9	15.4$\pm$0.3	43.0$\pm$1.9	31.9$\pm$3.6	**44.4$\pm$0.8**	26.4$\pm$2.8	32.2$\pm$2.1
LGG	ACC	63.1$\pm$0.9	62.7$\pm$2.1	62.7$\pm$2.4	64.2$\pm$0.1	64.8$\pm$0.2	63.9$\pm$0.3	63.8$\pm$0.4	64.7$\pm$0.1	62.4$\pm$0.8	**67.7$\pm$1.2**
	NMI	11.5$\pm$1.0	10.2$\pm$1.4	9.9$\pm$2.3	12.6$\pm$0.2	13.4$\pm$0.4	10.8$\pm$0.4	12.0$\pm$1.1	11.6$\pm$0.2	10.9$\pm$0.5	**13.7$\pm$2.0**
	ARI	7.8$\pm$1.0	6.5$\pm$2.0	7.4$\pm$2.5	7.9$\pm$0.1	8.0$\pm$0.2	7.6$\pm$0.3	7.6$\pm$0.5	8.4$\pm$0.1	7.1$\pm$0.5	**10.1$\pm$1.3**
	ASW	51.4$\pm$2.5	48.3$\pm$3.2	48.5$\pm$3.0	20.2$\pm$0.1	31.4$\pm$0.7	53.9$\pm$3.5	55.3$\pm$3.6	**64.6$\pm$0.7**	29.2$\pm$0.4	61.0$\pm$2.7

**Table 2:** Clustering performance comparison of different methods across different single-cell multi-omics datasets. All results are reported as mean $\pm$ standard deviation over 10 independent runs, with values rounded to one decimal place. The best results are highlighted in bold.

		Method									
Dataset	Metric	AE	DAE	VAE	MOFA	MOSA	scMDC	Matilda	scMHNN	scMNMF	UDECMO
TEA	ACC	54.6$\pm$2.4	54.1$\pm$1.6	17.7$\pm$1.7	61.9$\pm$0.1	62.7$\pm$0.1	57.8$\pm$2.5	62.7$\pm$3.2	65.7$\pm$3.1	56.4$\pm$1.0	**80.3$\pm$1.6**
	NMI	46.7$\pm$1.1	48.3$\pm$0.3	1.5$\pm$0.7	64.3$\pm$0.0	69.4$\pm$0.0	54.5$\pm$3.2	61.2$\pm$4.5	65.3$\pm$0.8	62.6$\pm$1.6	**73.5$\pm$1.8**
	ARI	30.4$\pm$2.6	34.2$\pm$0.5	1.1$\pm$0.5	46.6$\pm$0.1	48.5$\pm$0.1	34.0$\pm$2.7	42.9$\pm$4.1	50.5$\pm$2.4	41.5$\pm$1.6	**64.8$\pm$2.2**
	ASW	32.1$\pm$1.3	28.5$\pm$0.5	22.9$\pm$6.7	21.6$\pm$0.0	25.4$\pm$0.0	49.7$\pm$1.4	38.4$\pm$2.5	34.2$\pm$1.6	34.6$\pm$1.0	**62.7$\pm$2.0**
DOGMA	ACC	30.8$\pm$2.1	36.5$\pm$2.4	17.4$\pm$2.1	32.1$\pm$1.4	28.5$\pm$0.4	37.9$\pm$0.8	40.3$\pm$3.1	35.0$\pm$0.3	31.9$\pm$0.8	**46.2$\pm$2.6**
	NMI	30.9$\pm$1.8	32.6$\pm$1.3	21.1$\pm$3.3	36.4$\pm$0.3	32.3$\pm$0.3	35.6$\pm$0.7	37.6$\pm$2.8	38.9$\pm$0.2	33.6$\pm$1.8	**44.8$\pm$3.6**
	ARI	10.1$\pm$1.5	16.3$\pm$1.2	7.8$\pm$1.7	21.6$\pm$1.1	20.9$\pm$0.5	20.6$\pm$1.0	24.2$\pm$2.5	34.8$\pm$0.4	23.2$\pm$1.3	**39.9$\pm$2.7**
	ASW	10.8$\pm$1.2	11.3$\pm$0.7	5.3$\pm$0.5	15.2$\pm$0.3	13.5$\pm$0.4	18.1$\pm$1.3	15.4$\pm$2.6	27.8$\pm$1.4	15.8$\pm$0.8	**28.6$\pm$3.1**
NEAT	ACC	32.1$\pm$4.5	35.8$\pm$3.7	25.2$\pm$2.7	30.7$\pm$0.2	32.9$\pm$0.2	34.9$\pm$2.0	40.8$\pm$2.8	34.7$\pm$1.5	36.5$\pm$1.5	**48.8$\pm$2.7**
	NMI	10.0$\pm$4.7	16.7$\pm$2.9	0.9$\pm$1.0	8.8$\pm$0.2	11.5$\pm$0.3	16.2$\pm$2.2	20.2$\pm$3.3	13.8$\pm$4.2	15.4$\pm$1.6	**30.2$\pm$3.1**
	ARI	6.4$\pm$3.9	12.5$\pm$3.6	0.6$\pm$0.7	3.5$\pm$0.0	5.7$\pm$0.1	6.2$\pm$1.0	15.1$\pm$3.2	13.1$\pm$2.1	14.5$\pm$1.3	**19.5$\pm$3.4**
	ASW	24.0$\pm$5.9	12.1$\pm$0.3	27.5$\pm$6.5	13.5$\pm$0.1	14.5$\pm$0.2	25.6$\pm$2.7	24.6$\pm$4.9	30.4$\pm$5.4	20.2$\pm$5.0	**31.0$\pm$4.2**

For bulk multi-omics datasets, UDEC-MO achieves the best ACC, NMI, and ARI on KIPAN, BRCA, and LGG. On KIPAN, e.g. UDEC-MO obtains 82.0% ACC, 51.9% NMI, and 55.6% ARI. Similar trends are observed on BRCA and LGG, indicating that incorporating UAR and uncertainty-guided clustering can improve label-level clustering consistency in heterogeneous cancer datasets. In terms of ASW, UDEC-MO achieves the best performance on KIPAN, while other methods such as scMHNN obtain higher ASW on BRCA and LGG. This difference is expected because ASW is an internal metric that primarily reflects geometric compactness and separation in the embedding space, rather than direct alignment with ground-truth labels. In this regard, UDEC-MO focuses more on improving label-level clustering consistency, without explicitly enforcing geometric structure, whereas contrastive learning-based methods such as scMHNN [35] are designed to enhance embedding quality, which may lead to higher ASW.

For single-cell multi-omics datasets, UDEC-MO shows more pronounced advantages. On TEA-seq, UDEC-MO achieves 80.3% ACC, 73.5% NMI, 64.8% ARI, and 62.7% ASW. Consistent improvements are also observed on DOGMA-seq and NEAT-seq, where UDEC-MO attains the best performance across all four metrics. These results suggest that uncertainty-aware representation learning and reliability-guided clustering are particularly effective for high-dimensional, sparse, and noisy tri-modal single-cell data.

Overall, these results indicate that explicitly modeling multi-level data uncertainty and incorporating instance-level reliability into the clustering objective can improve clustering robustness across both bulk and single-cell multi-omics settings.

### Ablation and convergence analysis

**Figure 3:**
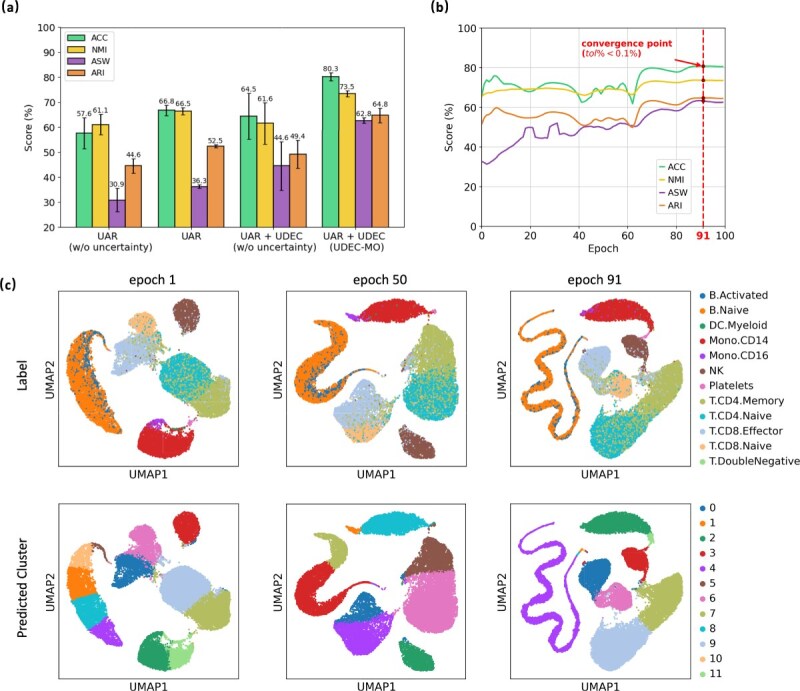
Ablation study and convergence analysis of UDEC-MO on TEA-seq dataset. (a) Ablation study comparing different model variants, including UAR (w/o uncertainty), UAR, UAR + UDEC (w/o uncertainty), and UAR + UDEC (i.e. the full UDEC-MO). The quality of multi-omics embeddings is evaluated using clustering metrics and presented as bar plots. (b) Convergence curves of clustering metrics during the UDEC optimization stage. (c) Visualization and quantitative evaluation of embedding evolution on the TEA-seq dataset at different DEC stages (epochs 1, 50, and 91), corresponding to early, intermediate, and converged states. UMAP visualizations are shown with both ground-truth cell-type labels and predicted cluster assignments, and the corresponding trend in panel (b) illustrates the progressive improvement of embedding quality.

To further investigate the contribution of different components in UDEC-MO and understand the optimization behavior of the clustering stage, we conduct ablation studies and convergence analysis on TEA-seq dataset, as illustrated in Fig. 3.

We first evaluate the impact of uncertainty modeling and uncertainty-guided clustering optimization by comparing four model variants: (i) UAR without uncertainty modeling, (ii) UAR with uncertainty modeling, (iii) UAR(w/o uncertainty) combined with standard DEC (i.e. UDEC without uncertainty-guided weighting), and (iv) the full model UDEC-MO with UDEC. As shown in Fig. 3(a), incorporating uncertainty modeling improves clustering performance and reduces variance, indicating enhanced robustness of the learned embeddings. In addition, introducing DEC further improves performance compared with reconstruction-only variants. However, directly applying standard DEC on embeddings learned without uncertainty modeling leads to less stable results. In contrast, jointly integrating UAR and uncertainty-guided clustering yields more consistent improvements. This suggests that uncertainty modeling not only improves representation quality but also provides more reliable guidance for clustering optimization.

We then analyze the convergence behavior during the UDEC optimization stage. As shown in Fig. 3(b), clustering metrics, including ACC, NMI, ARI, and ASW, exhibit an overall increasing trend and gradually stabilize as training proceeds. Although minor fluctuations are observed during optimization, the performance converges after $\sim$91 epochs, where the change ratio of cluster assignments falls below the predefined threshold ($0.1\%$), indicating stable clustering results.

To further illustrate how clustering optimization refines the learned representations, we visualize the embedding evolution on the TEA-seq dataset at different training stages (epochs 1, 50, and 91), as shown in Fig. 3(c). At the early stage, the embeddings are relatively dispersed with limited cluster separability. As optimization progresses, clusters become progressively more compact and better separated, and the predicted cluster assignments show improved alignment with ground-truth cell types. At convergence, the embedding space exhibits clearer cluster structures, with improvements observed in both geometric compactness (ASW) and label-level clustering metrics (ACC, NMI, and ARI).

Overall, these results indicate that UDEC-MO effectively improves clustering performance by combining uncertainty-aware representation learning with uncertainty-guided clustering optimization, leading to more stable and biologically meaningful multi-omics embeddings.

### Multi-level dynamic uncertainty analysis

In this section, we analyze the modeled multi-level dynamic uncertainty from the UAR stage, aiming to assess the model’s ability to capture uncertainty at multiple levels of multi-omics data, including feature-level, modality-level, and instance-level. Due to inherent biological variability and the technical challenges associated with data acquisition, both bulk and single-cell multi-omics data are often complex and noise-prone. Our multi-level dynamic uncertainty model estimates uncertainty at different granularities, thereby providing a more comprehensive characterization of data reliability and improving interpretability.

It is worth noting that, following prior uncertainty-aware representation learning and uncertainty quantification studies [39, 51], the estimated uncertainty in this work should be primarily interpreted as a reliability-related indicator under the learned reconstruction model, reflecting data uncertainty associated with data quality and noise characteristics. Therefore, the proposed uncertainty should be interpreted from the perspective of reliability assessment. At the same time, high uncertainty may also occur in biologically meaningful boundary or transitional states, especially in single-cell data.

#### Feature-level dynamic uncertainty analysis

We first evaluate the capability of UDEC-MO to capture feature-level dynamic uncertainty using the mRNA modality of the BRCA dataset. Considering that multi-omics data are affected by different types of noise in practice, we introduce two representative forms of controlled perturbations: Gaussian noise to simulate continuous measurement noise, and dropout noise to mimic sparsity and missing signals commonly observed in sequencing data. Specifically, Gaussian noise with increasing intensity from 0.0 to 4.0 and dropout noise with rates ranging from $p=0.1$ to $0.4$ are applied to the input features.

**Figure 4:**
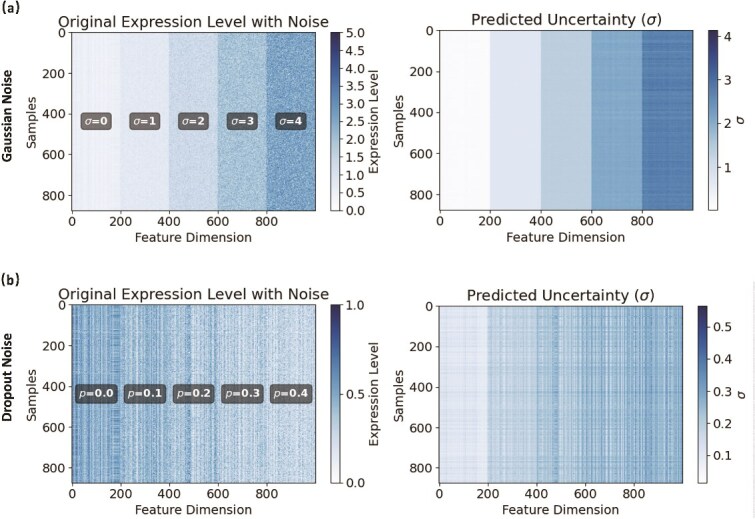
Feature-level uncertainty analysis on the mRNA modality of the BRCA dataset. (a) The left panel shows gene expression matrices with added Gaussian noise, where noise intensity increases from 0.0 to 4.0 in steps of 1. Each row represents a sample and each column a feature (1000 features across 865 samples), with color intensity indicating expression levels. The right panel shows the corresponding feature-level uncertainty predicted by the model, where color intensity reflects the uncertainty magnitude. (b) The left panel shows gene expression matrices with simulated dropout noise, where the dropout rate $p$ varies from 0.1 to 0.4. The right panel presents the corresponding uncertainty heatmaps. These results demonstrate that the proposed model can effectively capture dynamic feature-level uncertainty under different noise conditions.

As shown in Fig. 4, the predicted feature-level uncertainty exhibits clear correspondence with the injected noise patterns. Under Gaussian perturbation, regions with higher noise intensity are consistently assigned larger uncertainty values, indicating that the model is sensitive to gradual changes in signal corruption. Under dropout perturbation, features and samples with higher dropout rates also show elevated uncertainty, suggesting that the model can effectively capture sparsity-induced unreliability. Importantly, this behavior is consistently observed across different samples and noise levels, demonstrating that the uncertainty estimation is adaptive rather than fixed.

Overall, these results indicate that the estimated feature-level uncertainty reflects the reliability of observed signals under diverse noise conditions and can serve as a useful indicator for identifying noise-affected features in multi-omics data.

#### Modality-level dynamic uncertainty analysis

We further evaluate the capability of UDEC-MO to capture modality-level dynamic uncertainty using the TEA-seq dataset, which includes transcriptomic (RNA), proteomic (protein), and epigenomic (ATAC) modalities. To simulate heterogeneous data quality across modalities, we introduce controlled perturbations by varying both noise intensity and the proportion of corrupted samples within each modality.

**Figure 5:**
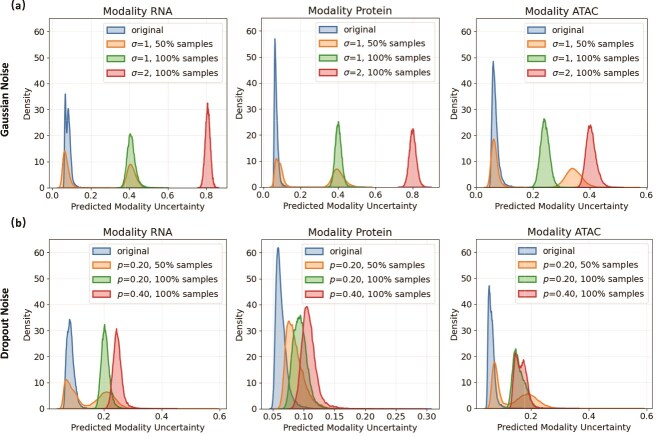
Modality-level uncertainty analysis on the TEA-seq dataset. (a) KDE plots of modality-level uncertainty distributions under Gaussian noise with varying noise intensity and different proportions of perturbed samples. (b) KDE plots of modality-level uncertainty distributions under dropout noise with varying dropout rates and sample proportions. Results are shown for the RNA (transcriptomic), PROTEIN (proteomic), and ATAC (epigenomic) modalities. These results demonstrate that the model can effectively capture dynamic modality-level uncertainty under different noise conditions.

Modality-level uncertainty is obtained by aggregating feature-level uncertainties within each modality. As shown in Fig. 5, the kernel density estimation (KDE) distributions reveal consistent trends across all modalities. Under both Gaussian and dropout perturbations, modality-level uncertainty increases as noise intensity or dropout rate increases, indicating that the model is sensitive to modality-specific degradation. Moreover, when a larger proportion of samples within a modality is corrupted, the overall uncertainty distribution shifts toward higher values, reflecting reduced reliability at the modality level. Importantly, these patterns are consistently observed across RNA, protein, and ATAC modalities, suggesting that the model can capture modality-level reliability differences in a stable and modality-agnostic manner.

Overall, these results demonstrate that the proposed uncertainty modeling framework effectively characterizes modality-level data quality and provides meaningful signals for multi-omics integration.

#### Instance-level dynamic uncertainty analysis

**Figure 6:**
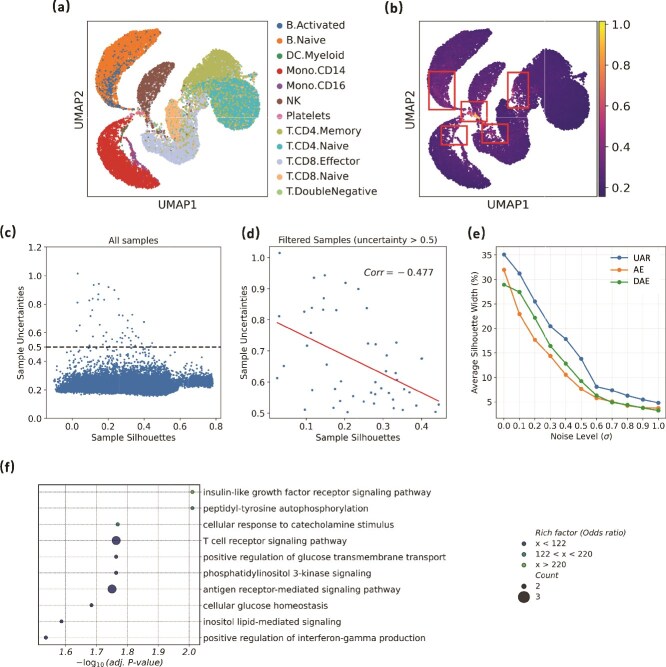
Instance-level uncertainty analysis on the TEA-seq dataset. (a) UMAP visualization of multi-omics embeddings generated by the UAR module, color-coded by cell type. (b) UMAP visualization of the same embeddings color-coded by instance-level uncertainty, where warmer colors indicate higher uncertainty. (c) Relationship between silhouette scores and instance-level uncertainty for all cells. (d) Relationship between silhouette scores and uncertainty among high-uncertainty cells with uncertainty >0.5, with the PCC reported. (e) ASW scores of embeddings under different noise levels, comparing UAR with AE and DAE. (f) GO Biological Process enrichment analysis of DEGs uniquely identified after removing the top 30% high-uncertainty cells in the T.CD4.Memory versus T.CD8.Effector comparison.

Finally, we investigate whether the proposed uncertainty modeling can capture reliability variation at the instance level. As shown in Fig. 6(a,b), cells located near the boundaries between cell-type clusters tend to exhibit higher uncertainty, suggesting that the estimated uncertainty can highlight ambiguous or less reliable cells in the embedding space.

To quantify this relationship, we examine the association between instance-level uncertainty and embedding quality using silhouette scores. As shown in Fig. 6(c), cells with higher uncertainty generally show lower silhouette scores, indicating weaker agreement with their corresponding cluster structures. When focusing on high-uncertainty cells with uncertainty >0.5, this trend becomes clearer, with a Pearson correlation coefficient (PCC) of $-0.477$ ($p=5.29\times 10^{-4}$), as shown in Fig. 6(d). These results suggest that instance-level uncertainty is closely associated with local embedding ambiguity and can serve as an indicator of sample reliability.

To evaluate robustness under noisy conditions, we compare UAR with AE and DAE by adding noise with varying intensities to the TEA-seq dataset and evaluating the resulting embeddings using ASW. As shown in Fig. 6(e), UAR consistently achieves higher ASW scores across different noise levels, indicating that uncertainty-aware modeling improves the robustness of multi-omics embeddings. DAE also shows better robustness than AE, further supporting the importance of noise-aware representation learning.

We then assess whether instance-level uncertainty can benefit downstream biological analysis. Using T.CD4.Memory and T.CD8.Effector cells as an example, we perform differential expression analysis before and after removing the top 30% high-uncertainty cells. As shown in Supplementary Fig. S1, uncertainty filtering leads to a refined set of DEGs under the same selection criteria. GO enrichment analysis of DEGs uniquely identified after filtering reveals several immune-related processes, including T cell receptor signaling pathway, antigen receptor-mediated signaling pathway, and positive regulation of interferon-gamma production, as shown in Fig. 6(f). These results provide supportive evidence that instance-level uncertainty can help interpret ambiguous cells and downstream signals, while high-uncertainty cells should not be regarded simply as technical noise, as they may also include boundary-like or transitional cells.

Overall, these analyses demonstrate that instance-level uncertainty not only reflects local embedding ambiguity but also provides a useful reliability measure for downstream analysis. By identifying ambiguous or low-reliability cells, UDEC-MO can improve the robustness and interpretability of multi-omics integration.

## Conclusions

In this study, we present UDEC-MO, an uncertainty-guided DEC framework for bulk and single-cell multi-omics data. UDEC-MO integrates UAR with reliability-weighted clustering optimization to improve clustering performance. Specifically, modality-specific encoders and an attention-based fusion network are first used to obtain a shared representation across omics modalities. The UAR stage then estimates feature-wise heteroscedastic uncertainty and aggregates it into modality-level and instance-level uncertainty scores, enabling multi-level characterization of data reliability. Based on the estimated instance-level uncertainty, the clustering stage assigns reliability weights to samples or cells and incorporates them into the DEC objective, reducing the influence of highly uncertain instances during cluster refinement.

Experiments on both bulk cancer multi-omics datasets and single-cell multi-omics datasets show that UDEC-MO achieves competitive or superior clustering performance compared with representative baseline methods. Additional analyses demonstrate that the estimated uncertainty is associated with feature-level noise, modality-level reliability variation, and instance-level embedding ambiguity. These results suggest that uncertainty modeling can provide useful reliability information for multi-omics integration and downstream interpretation, rather than serving only as a *post hoc* diagnostic measure. Meanwhile, instance-level uncertainty estimates may be associated with boundary-like or transitional cells, and the application of uncertainty should therefore be considered together with biological context and quality control information. Future work will also further validate the stability of uncertainty estimates and their relationship with available QC metrics.

While UDEC-MO achieves strong performance on label-based clustering metrics, it still has limitations in geometric structure preservation. Since the model focuses on improving clustering consistency through uncertainty-guided optimization, it does not explicitly enforce geometric compactness or separation in the embedding space. As a result, UDEC-MO may not always achieve the best performance on internal metrics such as ASW, reflecting a trade-off between clustering-oriented objectives and geometry-oriented embedding regularization. Future work will explore incorporating geometric constraints or contrastive objectives to further improve the structural quality of learned embeddings.

In addition, we plan to extend UDEC-MO to more complex multi-omics scenarios, including larger-scale single-cell atlases, datasets with missing modalities, and longitudinal disease studies, while improving its computational efficiency and scalability for large and heterogeneous biological datasets.

**Key Points**

- We propose UDEC-MO, an uncertainty-guided deep embedded clustering framework for robust bulk and single-cell multi-omics clustering.
- UDEC-MO estimates feature-wise heteroscedastic uncertainty through UAR and aggregates it into modality-level and instance-level uncertainty scores.
- Instance-level uncertainty is incorporated into UDEC objective as reliability weights, reducing the influence of highly uncertain samples or cells during cluster refinement.
- Experiments on bulk and single-cell multi-omics datasets demonstrate that UDEC-MO achieves competitive clustering performance and provides interpretable uncertainty estimates for identifying unreliable features, less reliable modalities, and ambiguous instances.

## Supplementary Material

main_sup_bbag435

## Data Availability

The UDEC-MO algorithm is implemented in Python and is available at https://github.com/ljw-struggle/UDEC-MO. The processed datasets used in this study are also provided in the same repository. All original datasets analyzed in this study are publicly available. Omics data for LGG, KIPAN, and BRCA, together with the grade information of LGG patients, were obtained from The Cancer Genome Atlas Program (TCGA) through Broad GDAC Firehose (https://gdac.broadinstitute.org/). The DOGMA-seq dataset was downloaded from OSF (https://osf.io/6kr4v). The TEA-seq and NEAT-seq datasets were obtained from the Gene Expression Omnibus (GEO) under accession numbers GSE158013 and GSE178707, respectively.
