# Supplementary material for: UDEC-MO: an uncertainty-guided deep embedded clustering framework for bulk and single-cell multi-omics data
Source: Brief Bioinform. 2026 Aug 10;27(4):bbag435. doi: 10.1093/bib/bbag435 (PMC13455639; doi:10.1093/bib/bbag435)
Supplement: Supplementary file 1 [file main_sup_bbag435.pdf]

Supplementary Materials for UDEC-MO: An Uncertainty-Guided Deep Embedded Clustering Framework for Bulk and Single-Cell Multi-Omics Data

Jiawei Li[1,2], Taoyuan Ye[2], Yilang Xiao[2], Mengyuan Zhao[3], Limin Jiang[5], Shizhan Chen[1], Fei Guo[4,*], and Jijun Tang[2,3,*]

[1]School of Computer Science and Technology, College of Intelligence and Computing, Tianjin University, Tianjin, China
[2]Faculty of Computer Science and Control Engineering, Shenzhen University of Advanced Technology, Shenzhen, China
[3]Shenzhen Institute of Advanced Technology, Chinese Academy of Sciences, Shenzhen, China
[4]School of Computer Science and Engineering, Central South University, Changsha, China
[5]Department of Public Health Sciences, University of Miami, Miami, USA

[*]Corresponding authors: feiguo@csu.edu.cn; jj.tang@siat.ac.cn

Supplementary Materials

Supplementary Table S1: Summary of Bulk Multi-Omics Datasets

Dataset	Samples	Classes	Features (mRNA, meth, miRNA)	Processed Features (mRNA, meth, miRNA)
BRCA	875	5	20,531, 20,106, 503	1000, 1000, 503
KIPAN	658	3	20,531, 20,111, 445	2000, 2000, 445
LGG	510	2	20,531, 20,114, 548	2000, 2000, 548

Note. LGG: Grade 2 ($n = 246$), Grade 3 ($n = 264$); KIPAN: KICH ($n = 66$), KIRC ($n = 318$), KIRP ($n = 274$); BRCA: Normal-like ($n = 115$), Basal-like ($n = 131$), HER2-enriched ($n = 46$), Luminal A ($n = 436$), Luminal B ($n = 147$).

Supplementary Table S2: Summary of Single-Cell Multi-Omics Datasets

Dataset	Cells	Classes	Features (ATAC, RNA, PROTEIN)	Processed Features (ATAC, RNA, PROTEIN)
TEA	25,517	12	128,853, 36,601, 47	30, 50, 47
DOGMA	13,763	25	68,963, 36,495, 210	30, 50, 210
NEAT	8,472	7	80,953, 36,601, 7	100, 100, 7

Note. TEA-seq contains human PBMCs with 12 immune-related cell types; DOGMA-seq contains human PBMCs with 25 cell types; NEAT-seq contains human CD4 memory T cells with 7 T cell subtypes.

Supplementary Algorithm S1: Training procedure of UDEC-MO

```
Input: Multi-omics data {X^(m)}_{m=1}^M, number of clusters C
Output: Embedding matrix H and cluster assignments y
Initialize modality-specific encoders, attention-based fusion network, reconstruction networks, and
  uncertainty prediction networks;
Stage 1: Uncertainty-aware reconstruction;
for each pretraining epoch do
    Obtain fused embeddings H from the multi-omics fusion module;
    Reconstruct each modality X̂^(m);
    Update model parameters using the mean squared reconstruction objective;
for each uncertainty-aware reconstruction epoch do
    Predict reconstruction means μ^(m) and log-variances s^(m);
    Update model parameters by minimizing L_UAR;
Compute feature-level uncertainty σ_ig^(m), modality-level uncertainty σ_i^(m), and instance-level uncertainty σ_i;
Stage 2: Uncertainty-guided DEC optimization;
Initialize cluster centroids U using k-means on H;
for each clustering epoch do
    Compute soft assignment matrix Q using Student's t-distribution;
    Update the auxiliary target distribution P;
    Compute reliability weights w_i = exp(−σ_i/τ) / Σ_{i'} exp(−σ_{i'}/τ), where τ = 1;
    Update the embedding network and cluster centroids by minimizing L_UDEC;
    Stop if the assignment change ratio is below the predefined threshold;
Return final cluster assignments according to Q;
```

Supplementary Table S3: Detailed descriptions of baseline methods used for comparison.

**Methods**	**Method descriptions**
AE	AE is a reconstruction-based embedding method. In our experiments, it uses modality-specific encoders to project different omics modalities into a shared latent representation and reconstructs each modality with corresponding decoders.
DAE	DAE extends AE by introducing stochastic corruption to the input data (e.g., masking or Gaussian noise) and learning to reconstruct the original clean multi-omics profiles. This denoising mechanism encourages the model to learn more robust latent representations that are less sensitive to noise.
VAE	VAE extends AE into a probabilistic generative framework by modeling latent variables as distributions and learning them via variational inference, enabling the model to capture uncertainty and variability in the latent space.
MOFA [1, 2]	MOFA is a probabilistic factor analysis framework for multi-omics integration. It learns interpretable low-dimensional factors that capture shared and modality-specific sources of variation across omics modalities, and the learned factors are used for downstream clustering.
MOSA [3]	MOSA is a deep generative multi-omics integration method that synthesizes missing molecular profiles to improve the completeness of multi-omics representations. The resulting integrated representations are used for downstream clustering evaluation.
scMDC [4]	scMDC is a deep clustering method for single-cell multi-omics data. It combines ZINB-based representation learning with clustering optimization to jointly learn latent embeddings and cell clusters.
Matilda [5]	Matilda is a multi-task deep learning framework for multimodal single-cell omics. It integrates representation learning with auxiliary tasks such as data simulation, dimensionality reduction, classification, and feature selection.
scMHNN [6]	scMHNN is a hypergraph neural network method for single-cell tri-modal omics integration. It models high-order cross-modal relationships through hypergraph learning and contrastive learning, and uses the learned embeddings for clustering evaluation.
scMNMF [7]	scMNMF is a joint non-negative matrix factorization method for single-cell multi-omics clustering. It learns a shared cell-factor matrix and modality-specific loading matrices, coupling clustering with feature selection in an interpretable framework.
Cobolt [8]	Cobolt is a probabilistic generative framework for multimodal single-cell sequencing data. It learns a shared latent representation across modalities and can integrate paired multimodal measurements with single-modality reference datasets for downstream clustering and visualization.
scGLUE [9]	scGLUE is a graph-linked unified embedding method for heterogeneous single-cell multi-omics integration. It explicitly incorporates prior regulatory relationships between omics features and learns a common cell embedding for integration, clustering, and regulatory inference.
Mowgli [10]	Mowgli is a paired single-cell multi-omics integration method based on integrative non-negative matrix factorization and optimal transport. It jointly learns interpretable cross-modality factors and integrated cell representations for clustering analysis.
MultiMAP [11]	MultiMAP is a multimodal dimensionality reduction and integration method that extends manifold learning to multiple datasets or modalities. It constructs a joint low-dimensional embedding to preserve dataset-specific structure while aligning cells across modalities.

Note: For AE, DAE, and VAE, clustering results are obtained by applying k-means to the learned latent embeddings. For the remaining baseline methods, clustering results are obtained using the clustering procedures described in their original papers.

Supplementary Table S4: Additional benchmark results for recently evaluated methods across bulk and single-cell multi-omics datasets. ACC, NMI, ARI, and ASW are reported as mean ± standard deviation in percentages.

Bulk multi-omics datasets						Single-cell multi-omics datasets					
		Method						Method			
Dataset	Metric	Cobolt	scGLUE	Mowgli	MultiMAP	Dataset	Metric	Cobolt	scGLUE	Mowgli	MultiMAP
KIPAN	ACC	35.8±0.2	74.3±8.9	50.6±5.5	79.5±0.1	TEA	ACC	15.0±0.4	58.1±5.1	43.1±1.2	65.6±0.1
	NMI	1.2±0.1	38.9±9.6	9.9±5.0	51.0±0.1		NMI	1.1±0.1	61.5±3.7	52.3±0.9	66.5±0.0
	ARI	1.5±0.2	42.1±7.8	7.0±7.7	54.8±0.1		ARI	0.1±0.1	45.1±4.5	31.5±0.7	50.4±0.1
	ASW	47.4±1.7	26.5±8.4	47.4±8.9	54.0±0.3		ASW	35.0±0.6	26.2±7.5	41.4±1.3	39.7±0.1
BRCA	ACC	24.3±0.4	46.7±4.5	53.8±5.9	55.1±2.5	DOGMA	ACC	11.4±0.1	31.4±1.4	29.9±7.0	33.8±0.5
	NMI	0.6±0.1	25.5±8.5	21.8±6.1	40.1±3.0		NMI	0.9±0.0	40.8±1.3	22.8±3.4	43.6±0.2
	ARI	0.1±0.1	19.0±5.7	19.1±5.7	28.6±0.9		ARI	0.1±0.1	20.7±2.7	13.2±4.2	21.5±0.5
	ASW	43.0±1.3	20.9±7.4	70.2±8.2	45.5±2.0		ASW	23.9±0.6	22.5±1.7	79.2±8.6	31.5±0.5
LGG	ACC	51.2±0.7	58.5±4.0	57.5±4.6	58.8±0.3	NEAT	ACC	19.2±1.8	23.8±1.4	24.8±3.1	46.0±0.1
	NMI	0.1±0.0	4.4±4.2	4.2±4.1	2.2±0.1		NMI	1.1±0.3	1.4±0.2	3.6±2.4	30.1±0.1
	ARI	0.2±0.1	3.4±2.4	2.7±2.5	2.9±0.2		ARI	1.2±0.2	1.4±0.4	1.9±2.2	18.8±0.1
	ASW	36.3±1.1	27.0±9.4	49.4±9.9	44.2±0.8		ASW	7.7±3.7	42.9±5.4	81.7±6.7	14.3±0.0

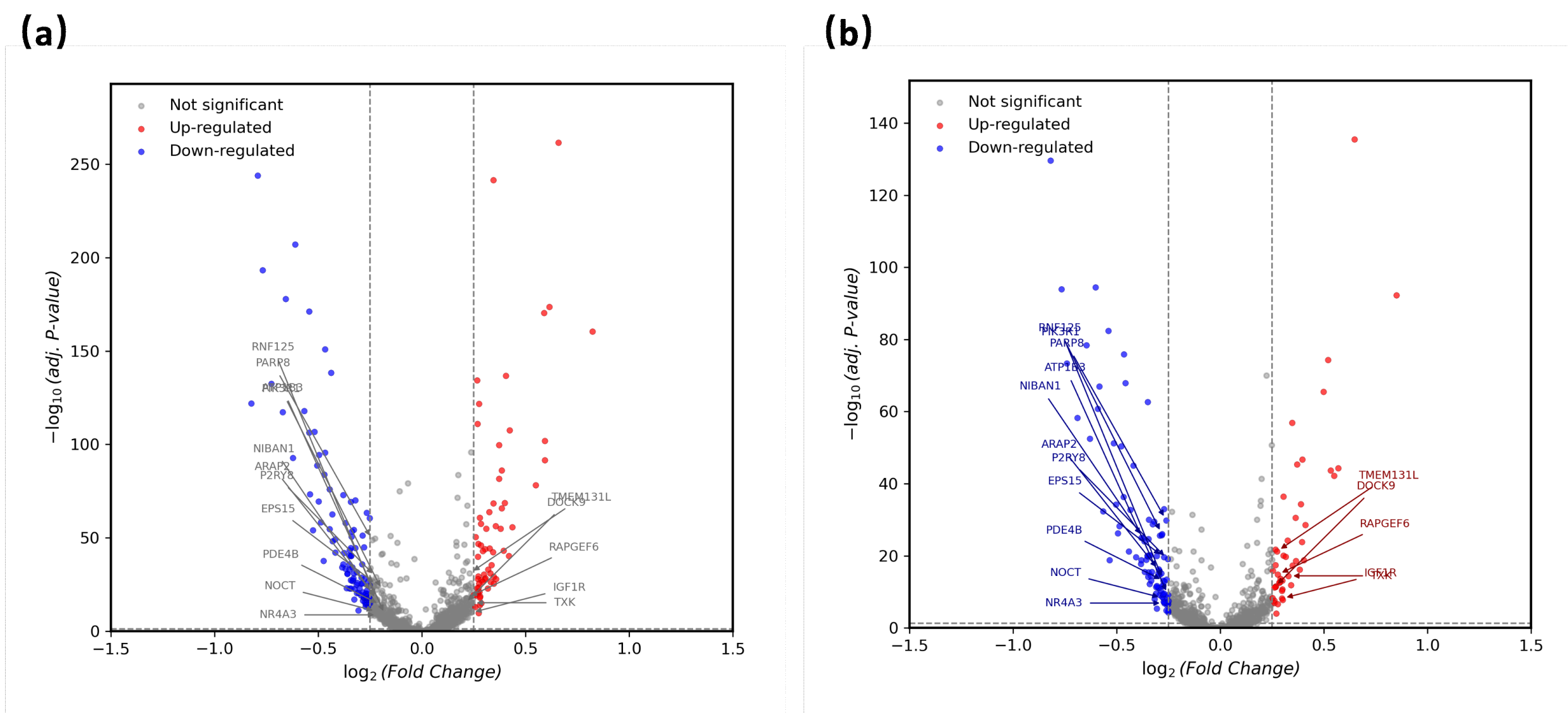

Supplementary Figure S1: Differential expression analysis between T.CD4.Memory and T.CD8.Effector cells in the TEA-seq dataset. (a) Volcano plot based on all cells. (b) Volcano plot after removing the top 30% high-uncertainty cells, with newly identified DEGs after uncertainty filtering annotated. Each point represents a gene, with colors indicating differential expression relative to T.CD8.Effector as the reference group: red, up-regulated in T.CD4.Memory; blue, down-regulated in T.CD4.Memory; and gray, not significant. Significant DEGs are defined using adjusted p-value < 0.05 and $|\log_2 \mathrm{FC}| > 0.25$. The x-axis shows $\log_2$ fold change, and the y-axis shows $-\log_{10}$(adjusted p-value).

Supplementary Legends

Supplementary Table S1. Summary of bulk multi-omics datasets, including sample numbers, class numbers, original feature dimensions, and processed feature dimensions for mRNA, DNA methylation, and miRNA modalities.
Supplementary Table S2. Summary of single-cell multi-omics datasets, including cell numbers, class numbers, original feature dimensions, and processed feature dimensions for ATAC, RNA, and protein modalities.
Supplementary Table S3. Detailed descriptions of the baseline methods used for comparison, including reconstruction-based models, probabilistic factor models, deep clustering methods, graph-based methods, and recently evaluated single-cell multi-omics integration methods.
Supplementary Table S4. Additional benchmark results for Cobolt, scGLUE, Mowgli, and MultiMAP across bulk and single-cell multi-omics datasets, reporting ACC, NMI, ARI, and ASW as mean $\pm$ standard deviation in percentages.
Supplementary Algorithm S1. Training procedure of UDEC-MO, including uncertainty-aware reconstruction, multi-level uncertainty estimation, reliability-weighted deep embedded clustering, and stopping criterion.
Supplementary Figure S1. Differential expression analysis between T.CD4.Memory and T.CD8.Effector cells in the TEA-seq dataset before and after removing the top 30% high-uncertainty cells.

References

[1] Ricard Argelaguet, Britta Velten, Damien Arnol, Sascha Dietrich, Thorsten Zenz, John C Marioni, Florian Buettner, Wolfgang Huber, and Oliver Stegle. Multi-omics factor analysis—a framework for unsupervised integration of multi-omics data sets. *Molecular systems biology*, 14(6):MSB178124, 2018.

[2] Ricard Argelaguet, Damien Arnol, Danila Bredikhin, Yonatan Deloro, Britta Velten, John C Marioni, and Oliver Stegle. Mofa+: a statistical framework for comprehensive integration of multi-modal single-cell data. *Genome biology*, 21(1):111, 2020.

[3] Zhaoxiang Cai, Sofia Apolinário, Ana R Baião, Clare Pacini, Miguel D Sousa, Susana Vinga, Roger R Reddel, Phillip J Robinson, Mathew J Garnett, Qing Zhong, et al. Synthetic augmentation of cancer cell line multi-omic datasets using unsupervised deep learning. *Nature communications*, 15(1):10390, 2024.

[4] Xiang Lin, Tian Tian, Zhi Wei, and Hakon Hakonarson. Clustering of single-cell multi-omics data with a multimodal deep learning method. *Nature communications*, 13(1):7705, 2022.

[5] Chunlei Liu, Hao Huang, and Pengyi Yang. Multi-task learning from multimodal single-cell omics with matilda. *Nucleic acids research*, 51(8):e45–e45, 2023.

[6] Wei Li, Bin Xiang, Fan Yang, Yu Rong, Yanbin Yin, Jianhua Yao, and Han Zhang. scmhnn: a novel hypergraph neural network for integrative analysis of single-cell epigenomic, transcriptomic and proteomic data. *Briefings in bioinformatics*, 24(6):bbad391, 2023.

[7] Yushan Qiu, Dong Guo, Pu Zhao, and Quan Zou. scmnmf: a novel method for single-cell multi-omics clustering based on matrix factorization. *Briefings in Bioinformatics*, 25(3):bbae228, 2024.

[8] Boying Gong, Yun Zhou, and Elizabeth Purdom. Cobolt: Integrative analysis of multimodal single-cell sequencing data. *Genome Biology*, 22(1):351, 2021. doi: 10.1186/s13059-021-02556-z.

[9] Zhi-Jie Cao and Ge Gao. Multi-omics single-cell data integration and regulatory inference with graph-linked embedding. *Nature Biotechnology*, 40(10):1458–1466, 2022. doi: 10.1038/s41587-022-01284-4.

[10] Geert-Jan Huizing, Ina Maria Deutschmann, Gabriel Peyré, and Laura Cantini. Paired single-cell Multi-omics data integration with Mowgli. *Nature Communications*, 14(1):7711, 2023. doi: 10.1038/s41467-023-43019-2.

[11] Mika Sarkin Jain, Krzysztof Polanski, Cecilia Dominguez Conde, Xi Chen, Jongeun Park, Lira Mamanova, Andrew Knights, Rachel A. Botting, Emily Stephenson, Muzlifah Haniffa, Austen Lamacraft, Mirjana Efremova, and Sarah A. Teichmann. MultiMAP: Dimensionality reduction and integration of multimodal data. *bioRxiv*, page 2021.02.16.431421, 2021. doi: 10.1101/2021.02.16.431421.
